# In Vitro and In Vivo Evaluation of *Bacillus* Strains as Prophylactic Agents Against Porcine Epidemic Diarrhea Virus

**DOI:** 10.3390/ani15040470

**Published:** 2025-02-07

**Authors:** You-Jia Chen, Chia-Fang Tsai, Chin-Wei Hsu, Hui-Wen Chang, Je-Ruei Liu

**Affiliations:** 1 Institute of Biotechnology, National Taiwan University, Taipei 10617, Taiwan; aria2929777@gmail.com (Y.-J.C.); r08642005@ntu.edu.tw (C.-F.T.); 2 School of Veterinary Medicine, National Taiwan University, Taipei 106319, Taiwan; cwihsu@ucdavis.edu (C.-W.H.); huiwenchang@ntu.edu.tw (H.-W.C.); 3 Department of Animal Science and Technology, National Taiwan University, Taipei 10617, Taiwan; 4 Bachelor Program of Biotechnology and Food Nutrition, National Taiwan University, Taipei 10617, Taiwan; 5 Center for Biotechnology, National Taiwan University, Taipei 10617, Taiwan; 6 Agricultural Biotechnology Research Center, Academia Sinica, Taipei 10617, Taiwan

**Keywords:** *Bacillus amyloliquefaciens*, *Bacillus licheniformis*, *Bacillus velezensis*, antiviral activity, porcine epidemic diarrhea virus

## Abstract

Porcine epidemic diarrhea virus (PEDV), particularly the highly virulent G2b strains, has inflicted substantial economic losses on the global swine industry. This study evaluated the prophylactic effects of three *Bacillus* strains—*B. amyloliquefaciens* LN, *B. licheniformis* CK, and *B. velezensis* AC—against PEDV infection using in vitro and in vivo models. In vitro experiments with Vero cells demonstrated that *B. amyloliquefaciens* LN increased cell viability, reduced PEDV-N expression, and modulated proinflammatory cytokine responses. In vivo, piglets supplemented with *B. amyloliquefaciens* LN exhibited alleviated diarrhea symptoms, suppression of fecal viral RNA shedding to below the detection limit, and restoration of gut microbiota balance by increasing Bacteroidetes and reducing Proteobacteria abundance. Mechanistic studies indicated that the measured interferon (IFN)-related genes were not significantly influenced in this study, suggesting that the protective effects of *B. amyloliquefaciens* LN may involve the modulation of inflammatory responses and the inhibition of viral replication through reduced PEDV-N expression. This study illustrates the potential of using *B. amyloliquefaciens* LN as a feed additive to prevent PEDV infection.

## Simple Summary

Porcine epidemic diarrhea is a devastating disease in pigs that causes severe diarrhea, dehydration, and high death rates, particularly in young piglets. This disease, caused by porcine epidemic diarrhea virus, has resulted in significant economic losses for pig farmers worldwide. Vaccines are not always effective, especially against newer virus strains, creating a need for alternative solutions. In this study, we tested three *Bacillus* strains to see if they could help prevent the disease. Among them, *Bacillus amyloliquefaciens* LN showed the best results. In the in vivo tests, it protected cells by reducing virus levels and controlling harmful inflammation. When given to piglets in their feed, this bacterial strain reduced diarrhea, completely stopped the spread of the virus through feces, and improved the balance of gut bacteria, which is essential for pig health. These findings suggest that adding *B. amyloliquefaciens* LN to pig feed could be a natural and effective way to protect pigs from this disease.

## 1. Introduction

Porcine epidemic diarrhea virus (PEDV), a member of the *Alphacoronavirus* genus within the *Coronaviridae* family, is a highly contagious virus responsible for porcine epidemic diarrhea (PED) [1]. Clinical manifestations of PEDV infection include acute gastrointestinal symptoms, such as anorexia, vomiting, watery diarrhea, and dehydration, with histological examinations typically revealing villous atrophy in the small intestines [2]. Initially identified in the United Kingdom in 1971, PEDV has primarily impacted swine industries across Asia [3]. Since 2010, the rise of the highly virulent and deadly PEDV strains has drawn significant global attention to the virus [4]. PEDV infects swine across all age groups, with outbreaks reaching nearly 100% prevalence and resulting in substantial economic losses for the swine industry. Although finishing pigs experience high morbidity with lower mortality rates, suckling piglets are disproportionately impacted, with mortality rates reaching 90–95% [4]. The emergence of the highly virulent PEDV G2 strain in southern China in 2010 led to devastating losses, with millions of piglets succumbing to the disease, even in vaccinated herds [5]. A similar strain surfaced in North America in 2013, resulting in the deaths of over 7 million piglets and a 10% reduction in the U.S. pig population [6].

To combat PEDV, strict biosecurity measures and vaccination are considered crucial. Vaccination remains the most effective strategy to mitigate losses. Current commercial options include inactivated and live attenuated vaccines, and innovative strategies, such as viral subunit vaccines, DNA vaccines, and viral replicating particle vaccines, have been developed in response to the variability of the virus [7]. The primary approach involves immunizing sows and gilts at the end of gestation to induce passive lactogenic immunity, thereby protecting piglets through maternal antibodies in colostrum and milk [7]. However, the rapid evolution of PEDV reduces vaccine efficacy against the emerging strains. Currently, no effective vaccines or antiviral drugs exist against the highly virulent PEDV G2 strain [8]. PEDV also poses challenges due to subclinical infections in growing–finishing pigs, which serve as reservoirs of the virus. Without proper disinfection, these reservoirs perpetuate the virus cycle, exposing sows and gilts and increasing the risk of transmission in farrowing houses [4,9].

Probiotics have long been recognized for their health benefits in humans and animals. In swine, probiotics as a feed additive have demonstrated improvements in average daily feed intake, average daily gain, and feed conversion ratio (FCR) across growth stages [10]. Specific probiotics or their derivatives, such as *Bacillus licheniformis* [11], *B. subtilis* [12], *Ligilactobacillus agilis* [13], *L. salivarius* [13], *Leuconostoc mesenteroides* [14], and *Lactiplantibacillus plantarum* [15], have shown potential in preventing PEDV invasion in in vitro or in vivo studies through diverse mechanisms. For instance, certain probiotic strains have been observed to decrease the expression of proinflammatory cytokines, such as tumor necrosis factor-α (TNF-α) and interleukin (IL)-8, which are significantly elevated during PEDV infection [13]. Other strains, such as *L*. *mesenteroides*, have been reported to enhance the expression of Type I interferon (IFN)-dependent genes, including Myxovirus resistance 1 (Mx1) and interferon-stimulated gene 15 (ISG15), which may contribute to their protective effects against PEDV [14]. Among these probiotics, *Bacillus* species are particularly noteworthy for their robust survival traits. Their spore-forming ability enables them to withstand harsh environmental conditions, such as the high temperatures and mechanical pressures involved in feed pelleting, without losing viability. This resilience not only ensures their prolonged shelf life but maintains their biological activity during storage and delivery, making them practical and reliable candidates for use as feed additives in livestock production [16,17].

In this study, we assessed three soil-derived *Bacillus* strains—*B. amyloliquefaciens* LN, *B. licheniformis* CK, and *B. velezensis* AC—for their potential to support intestinal health and enhance host resilience against PEDV infection. The evaluation was conducted through a combination of in vitro and in vivo approaches.

## 2. Materials and Methods

### 2.1. Virus, Cell, and Bacterial Strains

The PEDV G2 strain (Taiwan Pintung 52 strain) utilized in this study was initially isolated in early 2014 from the intestinal homogenate of a 7-day-old piglet in Taiwan. This strain was subsequently adapted to the African green monkey kidney cell line Vero, following the protocol established by Chang et al. [18]. Infection and propagation of PEDV in Vero cells were conducted according to the methods described by Chang-Liao et al. [14]. Viral titration was conducted using the Reed–Müench method, as described by Chang-Liao et al. [14], with the fifty percent tissue culture infective dose (TCID_50_) calculated as the reciprocal of the highest virus dilution that induced cytopathic effects (CPE).

Vero cells, obtained from the Bioresource Collection and Research Center of the Food Industry Research and Development Institute (Hsinchu, Taiwan), were cultured in Dulbecco’s Modified Eagle’s Medium (DMEM; Gibco, Grand Island, NY, USA) supplemented with 10% fetal bovine serum (FBS; Moregate Biotech, Queensland, Australia). The cells were maintained at 37 °C in a humidified environment with 5% CO_2_ and 95% air.

Three *Bacillus* strains, *B. amyloliquefaciens* LN, *B. licheniformis* CK1, and *B. velezensis* AC, were isolated and identified as previously demonstrated [19,20,21]. The *Bacillus* strains were grown in Luria–Bertani (LB) broth (Difco Laboratories Inc., Detroit, MI, USA) at 37 °C for 16 h using an orbital shaker set at 200 rpm (Major Science Inc., Taipei, Taiwan). Bacterial concentrations were measured either by optical density at 600 nm or through traditional colony counting methods.

### 2.2. Cytotoxicity Assay of Bacillus Strains

Extracellular supernatants and cell pellets of the *Bacillus* strains were obtained from 1.0 mL aliquots of overnight cultures through centrifugation at 9000× *g* for 10 min at 4 °C. The resulting cell pellets were washed twice with phosphate-buffered saline (PBS; 0.1 M, pH 7.0), resuspended in 1.0 mL of PBS, and sonicated for 10 min using an ultrasonicator (Model XL, Misonix, Inc., Farmingdale, NY, USA). Following sonication, the samples were centrifuged again at 13,000× *g* for 20 min at 4 °C to separate intracellular extracts from cell-wall fractions.

The cytotoxicity of extracellular supernatants, intracellular extracts, and cell-wall fractions on Vero cells were assessed using the 3-(4,5-dimethylthiazol-2-yl)-2,5-diphenyltetrazolium bromide (MTT) assay [22]. In brief, 3.0 × 10^4^ Vero cells were seeded into 96-well plates in 100 μL of DMEM and incubated at 37 °C for 24 h. Subsequently, 100 μL of each *Bacillus* preparation was added to the wells, and the plates were incubated for an additional 24 h at 37 °C. After incubation, the cells were washed twice with PBS, followed by the addition of 100 μL of MTT (5 mg/mL in DMEM) to each well. The plates were incubated at 37 °C for 2 h to allow for formazan crystal formation. The medium was then removed, and 100 μL of dimethyl sulfoxide (DMSO) was added to each well to dissolve the crystals. Absorbance at 570 nm was recorded using a microplate reader (Victor3, PerkinElmer Inc., Waltham, MA, USA), and cell viability was determined using the following formula:

Cell viability (%) = absorbance of the sample/absorbance of the control × 100

### 2.3. Assessment of the Prophylactic Effects of Bacillus Strains Against PEDV

To evaluate the in vitro prophylactic effects of the *Bacillus* strains, intracellular extracts and cell-wall fractions were prepared as previously described, and PEDV preparations were adjusted to a titer of 200 TCID_50_/mL. Vero cells (3.0 × 10⁴) were seeded into 96-well plates with 100 μL of the modified post-inoculation (PI) medium containing DMEM, 0.3% tryptose phosphate broth, 0.02% yeast extract, and 10 μg/mL of trypsin. The plates were incubated at 37 °C for 24 h. Following incubation, the cells were pretreated for 24 h with 100 μL of either *Bacillus* intracellular extracts, cell-wall fractions, human interferon α2b (IFN-α2b; 600 IU; ProSpec-Tany TechnoGene Ltd., Ness Ziona, Israel) as a positive control, or PBS as a negative control. After pretreatment, the cells were washed twice with PBS and then inoculated with 200 μL of PI medium containing 200 TCID_50_/mL of PEDV (delivering a total viral load of 40 TCID_50_ per well) or PBS for mock-infected controls. The viral inoculum was removed after 1 h of incubation at 37 °C, and the cells were washed twice with PBS before adding 200 μL of fresh PI medium. Cell viability was assessed 48 h post-infection using the MTT assay.

### 2.4. Quantitative Reverse-Transcription (qRT-PCR) Analysis of PEDV-N, Type I IFN-Dependent Genes, and Proinflammatory Cytokines

The expression levels of the PEDV nucleocapsid protein (PEDV-N) were analyzed in both the intracellular fractions and culture supernatants of Vero cells. Additionally, Type I IFN-dependent genes (ISG15, Mx1, and OAS1) and proinflammatory cytokines (IL-1β, IL-6, IL-8, and TNF-α) were quantified from the intracellular fraction. Vero cells were pretreated for 24 h with *Bacillus* intracellular extracts, cell-wall fractions, human IFN-α2b, or PBS before being mock-infected or infected with PEDV for 1 h. Following viral removal and the addition of fresh PI medium, cells were incubated at 37 °C for 48 h. RNA was extracted using TRIzol reagent (Invitrogen, Carlsbad, CA, USA), and qRT-PCR was conducted to measure the mRNA expression of PEDV-N, Type I IFN-dependent genes, and proinflammatory cytokines. Gene expression levels were normalized to glyceraldehyde 3-phosphate dehydrogenase (GAPDH) using the comparative CT method. The primer sets used for qRT-PCR are detailed in Table 1.

### 2.5. Animal Feed Preparation and Piglet PEDV Infection

The overnight bacterial cultures of *B. amyloliquefaciens* LN and *B. velezensis* AC were centrifuged to remove extracellular supernatants, and the resulting cell pellets were freeze-dried to obtain bacterial powders, which were then mixed with a commercial artificial milk feed powder (Anyou Biotechnology, Shanghai, China) to achieve a final bacterial concentration of 10^8^ CFU/kg.

Twenty cross-bred male post-weaning piglets (Duroc × Large White, 3 weeks old) from a specific pathogen-free (SPF) farm were acclimatized for 1 week before the experiment. The piglets were maintained under controlled conditions (18–25 °C and 50–70% humidity) with a 12-h light/dark cycle in the Animal Resource Center at National Taiwan University. Following an acclimatization period, they were randomly assigned to four groups, with each group consisting of five animals: (1) mock infection (Mock); (2) PEDV infection (PEDV); (3) PEDV infection with *B. amyloliquefaciens* LN supplementation (PEDV+LN); (4) PEDV infection with *B. velezensis* AC supplementation (PEDV+AC). On the 7th day of the trail, piglets in the PEDV infection groups were orally inoculated with 5 mL of 10^5^ TCID_50_/mL PEDV in PI medium, while control piglets received saline. PEDV inoculation and preparation followed previously described methods [11]. Each group was housed in separate cages, and the Mock group placed in a separate room to avoid cross-infection. The experiment lasted for 3 weeks, during which time the animals had ad libitum access to diets and water. Body weight and feed intake were recorded weekly, while rectal swabs were collected daily to monitor viral shedding. Fecal scores were documented throughout this study. The experimental protocol was approved by the Institutional Animal Care and Use Committee of National Taiwan University (NTU108-EL-00009).

### 2.6. qRT-PCR Detection of Fecal Viral RNA

Fecal viral RNA shedding was detected and quantified using qRT-PCR. Rectal swabs were soaked in 900 μL of DPBS and mixed by vortexing to prepare a fecal suspension. Viral RNA was extracted from 100 μL of the suspension using the Cador Pathogen 96 QIAcube HT Kit (Qiagen Inc., Valencia, CA, USA), yielding 50 μL of RNA extract. cDNA was synthesized using the QuantiNova Reverse Transcription Kit (Qiagen). For qRT-PCR, 2 μL of RNA extract was used per reaction, and RNA copies were quantified as previously described [27]. The results were normalized to the original fecal suspension volume and expressed as log genomic equivalents/mL. The detection limit of the assay was 1.8 log genomic equivalents/mL, based on a PEDV RNA standard curve.

### 2.7. Metagenomic Analysis of Fecal Microbiota

Fecal samples were collected at 14 days post-infection (DPI) from three randomly selected piglets in each group. Genomic DNA was extracted from the fecal samples using the QIAamp Fast DNA Stool Mini Kit (Cat. 51604, Qiagen) following the manufacturer’s protocol. Microbiota analysis was conducted by BIOTOOLS Co., Ltd. (Taipei, Taiwan).

The hypervariable V3–V4 region of the bacterial 16S rRNA gene was amplified using the universal primers [26]. The initial PCR was performed with KAPA HiFi HotStart ReadyMix (Roche Applied Science, Indianapolis, IN, USA) under the following conditions: an initial denaturation at 95 °C for 3 min, followed by 25 cycles of 95 °C for 30 s, 55 °C for 30 s, and 72 °C for 30 s, and a final extension at 72 °C for 5 min. The PCR products were analyzed via electrophoresis, and DNA fragments approximately 500 bp in size were extracted and purified using AMPure XP beads (Beckman Coulter, Indianapolis, IN, USA). A secondary PCR was performed using the 16S rRNA V3–V4 amplicons with the Nextera XT Index Kit (Illumina, San Diego, CA, USA), which includes dual indices and Illumina sequencing adapters, to prepare the sequencing library. The library was sequenced on an Illumina MiSeq platform to generate paired-end 300-bp reads. Raw sequencing reads were assembled using FLASH v1.2.11, and low-quality reads were removed using the QIIME v1.9.1 pipeline [28]. Chimera-free sequences were generated with UCHIME, producing effective tags. Sequences with ≥97% identity were clustered into operational taxonomic units (OTUs) using the UPARSE algorithm [29] in the USEARCH v7.0.1090 pipeline [30]. Taxonomic annotation of representative sequences was conducted using the RDP Classifier v2.2 algorithm [31] with the Silva Database v132 [32]. To normalize sequencing depth across samples, OTU abundance data were rarefied to the minimum sequence depth using QIIME [28]. Alpha diversity, including the Shannon diversity index, was calculated to assess within-sample microbial diversity [33]. Beta diversity was evaluated using the Bray–Curtis dissimilarity and UniFrac distance metrics to compare the microbial community structures between groups. Principal coordinates analysis (PCoA) was used to visualize inter-sample differences [34]. All bioinformatic analyses were conducted with QIIME and custom R scripts [28].

### 2.8. Statistical Analysis

Statistical analyses were conducted using SPSS version 25 software (IBM SPSS, New York, NY, USA). Group differences were assessed using one-way analysis of variance (ANOVA), followed by Duncan’s multiple range test for post hoc comparisons. A *p*-value < 0.05 was considered statistically significant. Data are expressed as a mean ± standard deviation from three independent experiments.

## 3. Results

### 3.1. Cytotoxicity of Bacillus Strains on Vero Cells

Prior to evaluating the anti-PEDV potential of the *Bacillus* strains, the cytotoxicity of extracellular supernatants, intracellular extracts, and cell-wall fractions from *B. amyloliquefaciens* LN, *B. licheniformis* CK, and *B. velezensis* AC were assessed in Vero cells. The assay involved exposing Vero cells to 100 μL of each *Bacillus* preparation, prepared as detailed in the Section 2, for 24 h. The results indicated that intracellular extracts and cell-wall fractions from all three strains exhibited low cytotoxicity, maintaining cell viability above 80%. By contrast, extracellular supernatants displayed higher cytotoxicity, with cell viability dropping below 50%. Based on their minimal cytotoxicity under these conditions, intracellular extracts and cell-wall fractions were chosen for subsequent anti-PEDV experiments.

### 3.2. Prophylactic Effects of Bacillus Strains Against PEDV

**Figure 1:**
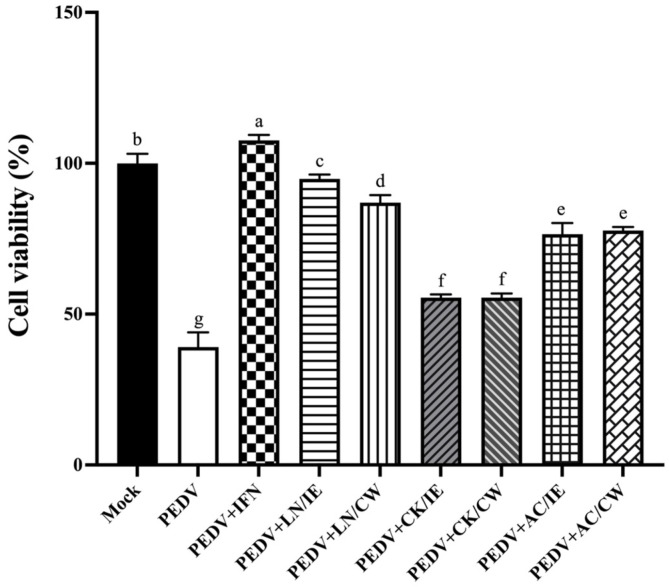
Protective effects of *Bacillus* intracellular extracts and cell-wall fractions against PEDV infection in Vero cells. The figure shows the prophylactic effects of intracellular extracts (IE) and cell-wall fractions (CW) from *Bacillus amyloliquefaciens* LN, *B*. *licheniformis* CK, and *B*. *velezensis* AC on Vero cells infected with porcine epidemic diarrhea virus (PEDV). Experimental groups included: Mock (PBS-pretreated, mock-infected), PEDV (PBS-pretreated, PEDV-infected), PEDV+IFN (IFN-α2b-pretreated, PEDV-infected), and PEDV+LN/IE, PEDV+LN/CW, PEDV+CK/IE, PEDV+CK/CW, PEDV+AC/IE, PEDV+AC/CW (pretreated with intracellular extracts or cell-wall fractions from LN, CK, or AC strains, followed by PEDV infection). Vero cells were pretreated with 100 μL of *Bacillus* preparations or IFN-α2b (6500 IU) for 24 h before infection with PEDV at a dose of 40 TCID_50_ per well. Data are expressed as a mean ± SD (*n* = 3). Bars sharing the same letter indicate no significant differences between groups (*p* > 0.05).

To assess the prophylactic potential of the *Bacillus* strains, Vero cells were pretreated with bacterial intracellular extracts or cell-wall fractions for 24 h prior to PEDV infection. IFN-α2b, used as a positive control, demonstrated the highest protective efficacy, as shown by a marked increase in cell viability (Figure 1). Pretreatment with *Bacillus* fractions also significantly increased cell viability compared to untreated PEDV-infected controls. Among the *Bacillus* strains, *B. amyloliquefaciens* LN provided the strongest prophylactic effect, followed by *B. velezensis* AC, while *B. licheniformis* CK showed the least activity. *B. amyloliquefaciens* LN and *B. velezensis* AC were selected for further mechanistic studies.

### 3.3. Effects on PEDV-N Protein Expression

**Figure 2:**
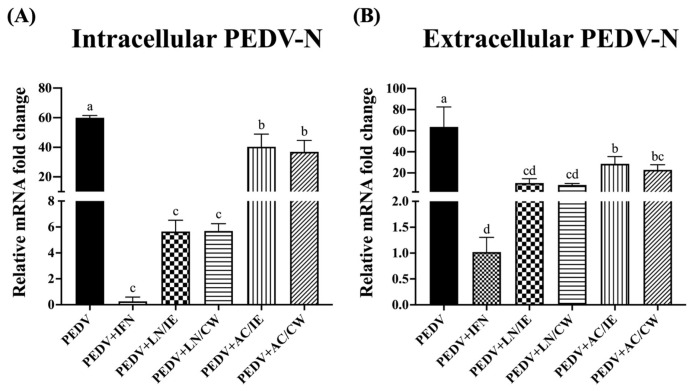
Impact of *Bacillus* intracellular extracts and cell-wall fractions on PEDV-N expression in Vero cells. The figure illustrates the effects of intracellular extracts (IE) and cell-wall fractions (CW) from *Bacillus amyloliquefaciens* LN and *B*. *velezensis* AC on the expression of the porcine epidemic diarrhea virus nucleocapsid (PEDV-N) protein in Vero cells. (**A**) Intracellular PEDV-N expression levels. (**B**) Extracellular PEDV-N expression levels. The experimental groups are consistent with those described in Figure 1. Data are presented as a mean ± SD (*n* = 3). Bars sharing the same letter indicate no significant differences between groups (*p* > 0.05).

To further confirm the anti-PEDV potentials of *B. amyloliquefaciens* LN and *B. velezensis* AC, PEDV-N expression levels were measured. As shown in Figure 2, pretreatment with intracellular extracts and cell-wall fractions from *B. amyloliquefaciens* LN and *B. velezensis* AC significantly reduced intracellular and extracellular expression levels of PEDV-N. As PEDV-N is the most abundant protein in the virion, our findings suggest that *B. amyloliquefaciens* LN and *B. velezensis* AC inhibit viral replication and particle release.

### 3.4. Effects on Type 1 IFN-Dependent Genes and Proinflammatory Cytokines

**Figure 3:**
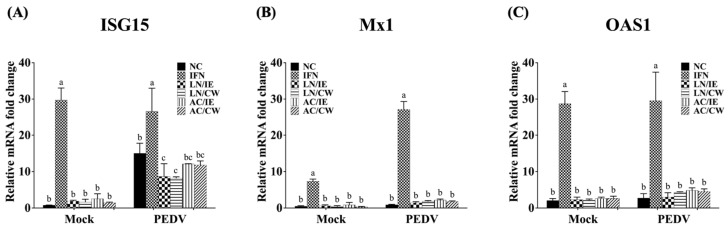
Effects of *Bacillus* intracellular extracts and cell-wall fractions on ISG expression in PEDV-infected Vero cells. The figure depicts the impact of intracellular extracts (IE) and cell-wall fractions (CW) from *Bacillus amyloliquefaciens* LN and *B*. *velezensis* AC on the expression levels of interferon-stimulated genes (ISGs) in Vero cells infected with porcine epidemic diarrhea virus (PEDV). (**A**) Interferon-stimulated gene 15 (ISG15). (**B**) Myxovirus resistance 1 (Mx1). (**C**) 2′-5′-Oligoadenylate synthetase 1 (OAS1). Groups: NC (PBS-pretreated), IFN (IFN-α2b-pretreated), LN/IE (intracellular extract of LN), LN/CW (cell-wall fraction of LN), AC/IE (intracellular extract of AC), and AC/CW (cell-wall fraction of AC). Data are shown as a mean ± SD (*n* = 3). Bars sharing the same letter indicate no significant differences between groups (*p* > 0.05).

**Figure 4:**
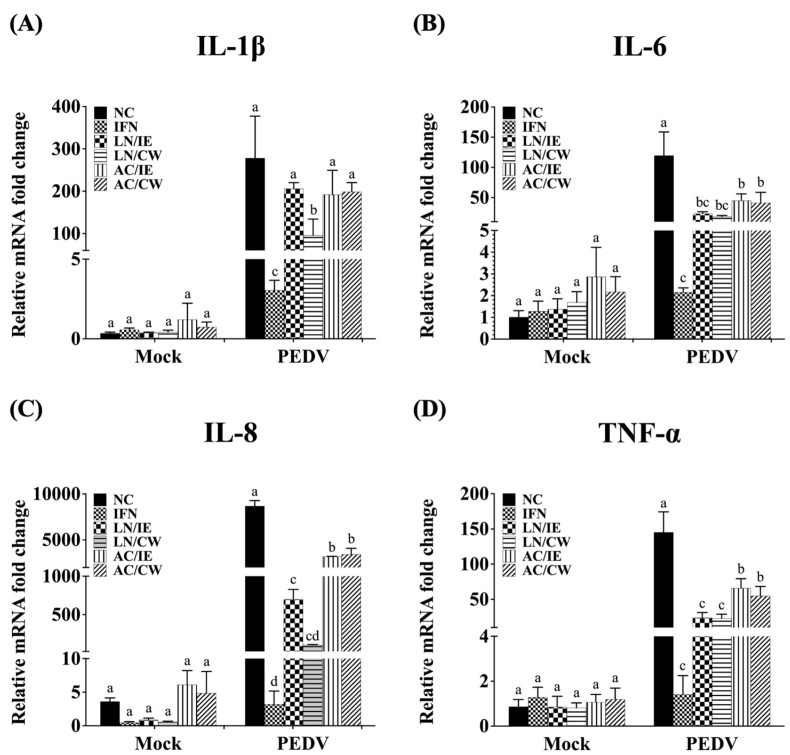
Effects of *Bacillus* intracellular extracts and cell-wall fractions on proinflammatory cytokine expression in PEDV-infected Vero cells. The figure illustrates the effects of intracellular extracts (IE) and cell-wall fractions (CW) from *Bacillus amyloliquefaciens* LN and *B*. *velezensis* AC on the expression levels of proinflammatory cytokines in Vero cells infected with porcine epidemic diarrhea virus (PEDV). (**A**) Interleukin (IL)-1β. (**B**) IL-6. (**C**) IL-8. (**D**) Tumor necrosis factor-α (TNF-α). Groups: NC (negative control, PBS-pretreated), IFN (IFN-α2b-pretreated), LN/IE (intracellular extract of LN), LN/CW (cell-wall fraction of LN), AC/IE (intracellular extract of AC), and AC/CW (cell-wall fraction of AC), as described in Figure 3. Data are presented as a mean ± SD (*n* = 3). Bars sharing the same letter indicate no significant differences between groups (*p* > 0.05).

To explore the molecular mechanisms underlying the prophylactic effects of *B. amyloliquefaciens* LN and *B. velezensis* AC against PEDV, the expression levels of type I IFN-dependent genes (including ISG15, Mx1, and OAS1), and proinflammatory cytokines (including IL-1β, IL-6, IL-8, and TNF-α) in Vero cells were quantified. Pretreatment with *Bacillus* fractions did not significantly alter the expression of type I IFN-dependent genes (ISG15, Mx1, OAS1) in either PEDV-infected or mock-infected cells (Figure 3). However, proinflammatory cytokines (IL-1β, IL-6, IL-8, and TNF-α) were significantly upregulated by PEDV infection but downregulated in *Bacillus*-treated groups (Figure 4). *B. amyloliquefaciens* LN showed stronger modulation of IL-8 and TNF-α compared to *B. velezensis* AC (Figure 4C,D).

### 3.5. Effects of Bacillus Supplementation on Body Weight and Clinical Scoring of Fecal Consistency of Piglets

To evaluate the efficacy of *Bacillus* in mitigating PEDV-induced diarrhea, 4-week-old piglets were orally administrated *B. amyloliquefaciens* LN or *B. velezensis* AC for 1 week prior to the PEDV challenge. Body weight and clinical symptoms were monitored daily for 14 days post-infection. Throughout the experimental period, the piglets in all groups exhibited steady weight gain, with no significant differences observed among the groups (Table 2).

**Figure 5:**
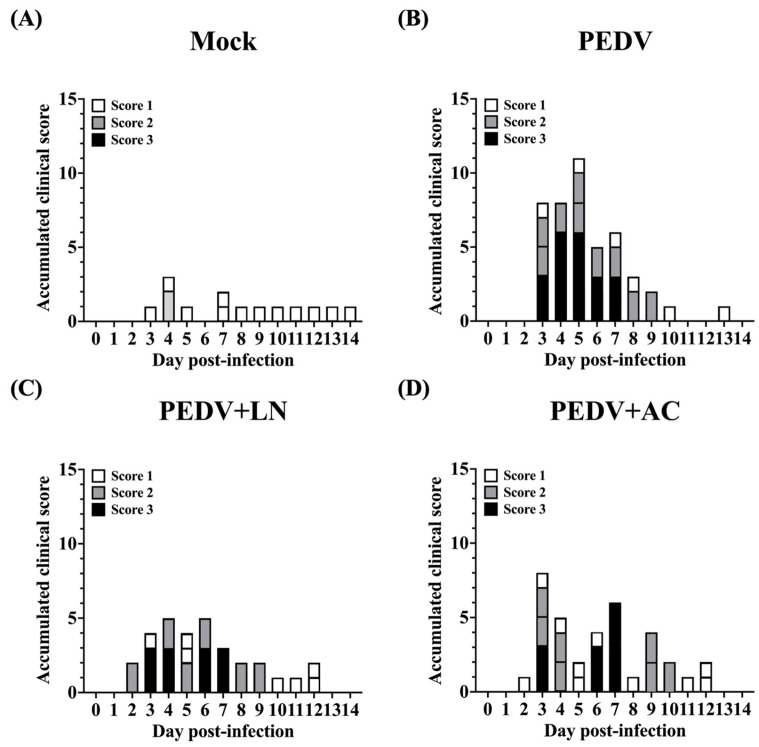
Effects of dietary supplementation with *Bacillus amyloliquefaciens* LN or *B. velezensis* AC on fecal consistency in porcine epidemic diarrhea virus (PEDV)-infected piglets. (**A**) Mock: mock-infected piglets. (**B**) PEDV: PEDV-infected piglets. (**C**) PEDV+LN: PEDV-infected piglets supplemented with *B. amyloliquefaciens* LN. (**D**) PEDV+AC: PEDV-infected piglets supplemented with *B. velezensis* AC. Fecal consistency was scored daily using a 4-point scale: 0 (normal), 1 (pasty feces), 2 (semi-fluid feces), 3 (watery diarrhea).

Fecal consistency was scored based on the following criteria: Score 0: normal clinical signs, well-formed feces; Score 1: pasty feces; Score 2: semi-fluid feces; Score 3: watery diarrhea. Before PEDV inoculation, all groups exhibited normal fecal consistency (Score 0, Figure 5). Following PEDV infection, piglets in the untreated PEDV group exhibited severe diarrhea, with fecal scores ranging from 2 (semi-fluid feces) to 3 (watery diarrhea) by 5 DPI (Figure 5B). Two out of five piglets in this group showed semi-fluid feces (Score 2), while the remaining three displayed watery diarrhea (Score 3). Piglets pretreated with *B. amyloliquefaciens* LN exhibited significantly milder symptoms. Most animals in this group maintained normal feces (Score 0) or pasty feces (Score 1) throughout the study period, with only one piglet experiencing transient watery diarrhea (Score 3) between 3 and 7 DPI (Figure 5C). Similarly, piglets in the *B. velezensis* AC group showed reduced diarrhea severity compared to the untreated PEDV group (Figure 5D), although symptoms were more pronounced than those observed in the *B. amyloliquefaciens* LN group.

### 3.6. Fecal Viral RNA Shedding

**Figure 6:**
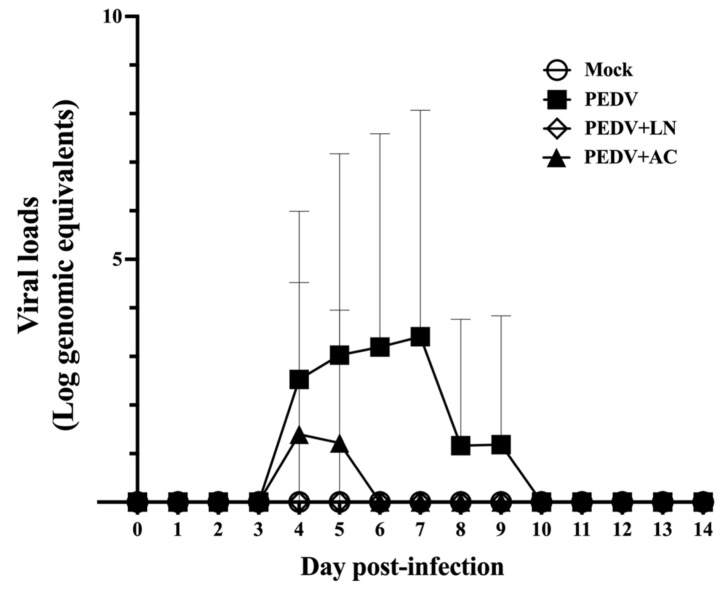
Effects of dietary supplementation with *Bacillus amyloliquefaciens* LN or *B. velezensis* AC on fecal viral shedding in porcine epidemic diarrhea virus (PEDV)-infected piglets. Groups: Mock (mock-infected piglets), PEDV (PEDV-infected piglets), PEDV+LN (PEDV-infected piglets supplemented with *B. amyloliquefaciens* LN), and PEDV+AC (PEDV-infected piglets supplemented with *B. velezensis* AC).

To assess the effects of *B. amyloliquefaciens* LN and *B. velezensis* AC on PEDV replication in vivo, fecal viral RNA shedding was monitored daily from 1 to 14 DPI using qRT-PCR. Viral RNA levels were quantified as log genomic equivalents/mL. As shown in Figure 6, the absence of viral RNA in the mock-infected control group confirmed the specificity of PEDV detection and the validity of the experimental design. In the PEDV-infected group, fecal viral RNA shedding was first detected at 4 DPI, with RNA levels peaking at 7 DPI and remaining detectable until 9 DPI (Figure 6). Piglets pretreated with *B. velezensis* AC showed significantly reduced fecal viral RNA shedding compared to the untreated PEDV group. Shedding was detected only on 4 and 5 DPI. Remarkably, no fecal viral RNA shedding was detected in piglets pretreated with *B. amyloliquefaciens* LN throughout the study period. This suppression of fecal viral RNA shedding to below the detection limit highlights the prophylactic efficacy of *B. amyloliquefaciens* LN in mitigating PEDV infection.

### 3.7. Fecal Microbiota Analysis

To examine the impact of *B. amyloliquefaciens* LN and *B. velezensis* AC supplementation on the fecal microbiota composition of PEDV-infected piglets, fecal samples were collected at 14 DPI and analyzed via 16S rRNA gene sequencing. The analysis yielded 926,112 high-quality reads, averaging 77,176 reads per sample (range: 66,786–93,638). These reads were grouped into 741 operational taxonomic units (OTUs) based on a 97% similarity threshold.

**Figure 7:**
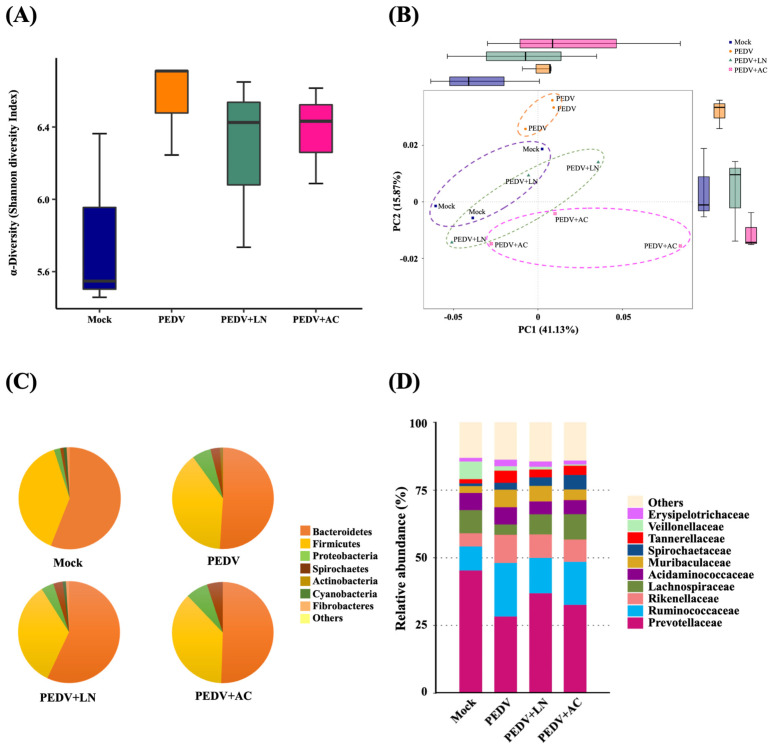
Effects of dietary supplementation with *Bacillus amyloliquefaciens* LN or *B. velezensis* AC on fecal microbiota in porcine epidemic diarrhea virus (PEDV)-infected piglets. (**A**) The Shannon diversity index showing alpha diversity. (**B**) Principal coordinates analysis (PCoA) of beta diversity based on the Bray–Curtis dissimilarity. (**C**) Relative abundance at the phylum level. (**D**) Relative abundance of dominant bacterial families. Groups: Mock (mock-infected piglets), PEDV (PEDV-infected piglets), PEDV+LN (PEDV-infected piglets supplemented with *B. amyloliquefaciens* LN), and PEDV+AC (PEDV-infected piglets supplemented with *B. velezensis* AC).

Alpha diversity, assessed through the Shannon diversity index, reflected microbial richness and evenness. Shannon diversity showed no significant differences between each group, but PEDV infection slightly increased the Shannon diversity compared to the mock-infected group, reflecting dysbiosis caused by the viral infection. In addition, supplementation with *B. amyloliquefaciens* LN or *B. velezensis* AC slightly reduced the Shannon diversity compared to the PEDV group, suggesting a stabilization of microbial communities (Figure 7A).

Beta diversity was evaluated using principal coordinates analysis (PCoA) based on the Bray–Curtis dissimilarity. The principal components accounted for a cumulative variance of 57%, with PC1 contributing 41.13% and PC2 contributing 15.87%. The PC1 versus PC2 plot revealed that the fecal microbiota compositions of PEDV-infected piglets clustered distinctly from those of mock-infected piglets, highlighting significant shifts in microbiota composition induced by PEDV infection. Piglets supplemented with *B. amyloliquefaciens* LN or *B. velezensis* AC displayed clustering closer to the mock group, suggesting partial restoration of microbiota composition (Figure 7B).

At the phylum level, Bacteroidetes and Firmicutes were the predominant taxa across all groups. In mock-infected piglets, Bacteroidetes comprised 56.1% of the microbial community, followed by Firmicutes (38.7%) and Proteobacteria (2.0%) (Figure 7C). PEDV infection reduced the relative abundance of Bacteroidetes to 51.0%, while increasing Proteobacteria to 5.8%, indicating microbiota dysbiosis. Supplementation with *B. amyloliquefaciens* LN increased the proportion of Bacteroidetes to 56.6% and reduced Proteobacteria to 3.1%. By contrast, *B. velezensis* AC supplementation led to a slight decrease in Bacteroidetes (50.1%) and a higher proportion of Proteobacteria (6.6%).

At the family level, PEDV infection disrupted the relative abundance of key bacterial families. *Prevotellaceae*, the most abundant family in mock-infected piglets (45.3%), decreased to 28.3% in PEDV-infected piglets. This decrease was partially reversed by *B. amyloliquefaciens* LN (36.9%) and *B. velezensis* AC (32.6%) supplementation. Similarly, the proportion of *Ruminococcaceae*, which increased in PEDV-infected piglets (19.8%) compared to the mock group (8.9%), was reduced to 13.1% and 15.9% by *B. amyloliquefaciens* LN and *B. velezensis* AC, respectively (Figure 7D).

## 4. Discussion

Since the 2010s, the highly virulent PEDV strains have caused devastating economic losses in the global swine industry. The G2 strains, including the lethal G2b variants, are now the predominant epidemic strains worldwide [6]. This study utilized the highly virulent G2b strain PEDV Pintung-52, isolated in Taiwan in 2014 [18], to evaluate the antiviral potential of *B. amyloliquefaciens* LN, *B. licheniformis* CK, and *B. velezensis* AC. Both in vitro and in vivo models demonstrated the superior efficacy of *B. amyloliquefaciens* LN in reducing viral replication, alleviating clinical symptoms, and restoring gut microbiota, making it a promising candidate for PEDV control.

The Vero cell line, commonly used in PEDV research, lacks the ability to produce type I IFNs due to a major deletion in the IFN gene cluster but retains functional IFN receptors and signaling pathways [35,36]. This characteristic makes them an ideal model for evaluating the impact of antiviral agents on viral replication and host cellular responses [37]. In this study, *B. amyloliquefaciens* LN demonstrated the strongest prophylactic effects among the tested strains, significantly increasing Vero cell viability and reducing PEDV-N expression levels. PEDV-N, as the nucleocapsid protein, plays a central role in viral replication and assembly, consistent with its structural and functional roles in coronaviruses. Moreover, PEDV-N has been shown to promote the acetylation and release of high mobility group box 1 (HMGB1), a key proinflammatory factor implicated in the pathogenesis of various inflammatory and autoimmune diseases [38]. The observed reduction in PEDV-N expression suggests that *B. amyloliquefaciens* LN may interfere with viral replication and HMGB1-mediated inflammatory responses, a mechanism that warrants further investigation.

PEDV infection is associated with excessive production of proinflammatory cytokines, including IL-1β, IL-6, IL-8, and TNF-α, leading to a “cytokine storm” that exacerbates tissue damage, immune cell death, and systemic inflammation [38]. These cytokines are major contributors to the pathogenesis of severe PEDV symptoms, such as diarrhea and intestinal epithelial damage. In this study, pretreatment with intracellular extracts or cell-wall fractions of *B. amyloliquefaciens* LN and *B. velezensis* AC significantly reduced proinflammatory cytokine expression in PEDV-infected Vero cells. The reduction in cytokine expression is likely linked to the observed suppression of PEDV-N levels, as PEDV-N promotes HMGB1 release, which in turn amplifies proinflammatory cytokine production and inflammatory damage [38]. Interestingly, IL-6 plays a dual role in immune responses by driving the differentiation of Th17 cells, which produce IL-17, and T follicular helper (Tfh) cells, which secrete IL-21. IL-17 primarily contributes to inflammatory responses, while IL-21 is crucial for germinal center formation, high-affinity antibody production, and durable humoral immunity, particularly against rapidly mutating viruses and emerging strains like PEDV G2. It has also been demonstrated that PEDV-infected CD103^+^dendritic cells (DCs) can promote the differentiation of CD4^+^T cells to T helper cells, including Th1, Tfh, and Treg cells [39]. Although the present study did not specifically evaluate the impact of *B*. *amyloliquefaciens* LN on antigen-specific IgA production or Tfh cell differentiation, these pathways represent promising areas for future research. Given the importance of Tfh cells in eliciting durable and potent neutralizing antibody responses, particularly against rapidly mutating viruses, future studies could explore the potential of *B*. *amyloliquefaciens* LN as an adjuvant alongside vaccines to potentiate Tfh-mediated immune responses. Such investigations should include evaluating neutralizing antibodies (NAbs), Tfh and Th17 cell responses, and cytokines, such as IL-21 and IL-17, to better understand the mechanisms underlying its effects.

PEDV has evolved sophisticated immune evasion strategies to suppress IFN production and signaling, limiting the induction of ISGs such as ISG15, Mx1, and OAS1 [35,40]. Consistent with these observations, PEDV infection in Vero cells did not significantly alter Mx1 or OAS1 expression levels, and pretreatment with *Bacillus* fractions failed to enhance these genes. While this study does not allow us to conclusively determine whether the effects of *B. amyloliquefaciens* LN and *B. velezensis* AC are independent of the type I IFN pathway, it is notable that the measured IFN-related genes were not significantly influenced. This observation could be due to an inadequate duration or dosing regimen. Alternatively, the observed effects may involve mechanisms such as modulating inflammatory responses, restoring intestinal barrier integrity, or promoting host resilience through microbiota-mediated pathways. Further studies are needed to elucidate these mechanisms, to optimize treatment conditions, and to identify the specific bioactive compounds involved.

In the in vivo study, supplementation with *B. amyloliquefaciens* LN significantly alleviated diarrhea severity, reduced fecal viral shedding, and restored gut microbiota composition in PEDV-infected piglets. PEDV infection disrupted gut microbiota by reducing the abundance of Bacteroidetes and increasing Proteobacteria, a hallmark of dysbiosis [41,42]. Supplementation with *B. amyloliquefaciens* LN effectively reversed these changes, increasing Bacteroidetes abundance and reducing Proteobacteria levels. Since an elevated prevalence of Proteobacteria is a diagnostic signature of dysbiosis and increased disease risk, *B. amyloliquefaciens* LN may enhance host digestive health by restoring microbiota balance. These findings align with previous studies highlighting the potential of probiotics to restore gut microbiota and improve intestinal health in PEDV-infected piglets [43].

Several studies have demonstrated the efficacy of probiotics in mitigating PEDV-associated symptoms. For instance, supplementation with *Limosilactobacillus reuteri* and *Lactobacillus amylovorus* improved survival rates, reduced weight loss, and mitigated intestinal damage in PEDV-infected piglets [44]. Similarly, *Lacticaseibacillus rhamnosus* GG enhanced intestinal morphology and reduced mucosal inflammation [43]. However, few studies have specifically addressed the role of probiotics in reversing PEDV-induced microbiota dysbiosis. This study uniquely demonstrates that *B. amyloliquefaciens* LN not only alleviates clinical symptoms but restores microbiota balance by increasing beneficial taxa and reducing dysbiosis-associated Proteobacteria. These findings highlight the ability of *B. amyloliquefaciens* LN to improve intestinal health and enhance host resilience to PEDV infection through its microbiota-modulating effects rather than acting as a direct antiviral agent.

## 5. Conclusions

This study demonstrated that *B. amyloliquefaciens* LN exhibits potent prophylactic effects against PEDV infection, as evidenced by its ability to increase cell viability, reduce PEDV-N expression, and suppress proinflammatory cytokines in in vitro Vero cell models. Furthermore, in vivo experiments revealed that supplementation with *B. amyloliquefaciens* LN alleviated diarrhea severity, suppressed fecal viral RNA shedding to below the detection limit, and restored gut microbiota balance by increasing the abundance of beneficial Bacteroidetes and reducing dysbiosis-associated Proteobacteria. According to these results, *B. amyloliquefaciens* LN has the potential to improve intestinal health and enhance overall host resilience against PEDV infection.

## Figures and Tables

**Table 1:** Primers used in this study.

Gene	Primer Sequence (5′ to 3′)	Reference
ISG15	Forward: GGGCAACGAGTTCCAGGT Reverse: CACCACCAGCAGGACCGT	[23]
MX1	Forward: GCAGCCAGTACGAGGAGAAG Reverse: CTCCTGACAGTGCCTCCAAC	[23]
OAS1	Forward: GGTTGTCTTCCTCAGTCCTC Reverse: AGCCTGGACCTCAAACTTCA	[23]
IL-1β	Forward: GCGGCAACGAGGATGACTT Reverse: TGGCTACAACAACTGACACGG	[24]
IL-6	Forward: TGTGAAAGCAGCAAAGAG Reverse: AGTGTCCTCATTGAATCCA	[24]
IL-8	Forward: GGAACCATCTCGCTCTGTGTAA Reverse: GGTCCACTCTCAATCACTCTCAG	[24]
TNF-α	Forward: GTGCTGGTGACTTTGGTGCTA Reverse: GAGAAGCCTCAGGTCCCAAT	[24]
PEDV-N	Forward: TGAGGGTGTTTTCTGGGTTG Reverse: TTGCCATTGCCACGACTC	[25]
GAPDH	Forward: AGCCAAAAGGGTCATCATCT Reverse: ATGAGTCCTTCCACGATACC	[23]
16S rRNA	Forward: CCTACGGGNGGCWGCAG Reverse: GACTACHVGGGTATCTAATCC	[26]

**Table 2:** Body weight (kg) of piglets at day post-infection (DPI) after PEDV.

Group	0 DPI	7 DPI	14 DPI
Mock ^1^	6.00 ± 2.36	9.10 ± 1.91	12.57 ± 3.06
PEDV	6.51 ± 1.30	10.17 ± 2.75	12.43 ± 2.02
PEDV+LN	7.00 ± 2.17	9.73 ± 2.63	11.93 ± 2.89
PEDV+AC	6.64 ± 1.23	11.00 ± 1.32	12.77 ± 1.89

^1^ Mock: mock-infected piglets. PEDV: PEDV-infected piglets. PEDV+LN: PEDV-infected piglets supplemented with *Bacillus amyloliquefaciens* LN. PEDV+AC: PEDV-infected piglets supplemented with *B. velezensis* AC.

## Data Availability

The data presented in this study are available on request from the corresponding author.
